# Geographical Patterns of HIV Sero-Discordancy in High HIV Prevalence Countries in Sub-Saharan Africa

**DOI:** 10.3390/ijerph13090865

**Published:** 2016-08-31

**Authors:** Diego F. Cuadros, Laith J. Abu-Raddad

**Affiliations:** 1 Department of Geography, University of Cincinnati, Cincinnati, OH 45221, USA; 2 Infectious Disease Epidemiology Group, Weill Cornell Medicine—Qatar, Cornell University, Qatar Foundation, Education City, Doha 24144, Qatar; lja2002@qatar-med.cornell.edu; 3 Department of Healthcare Policy and Research, Weill Cornell Medicine, Cornell University, New York, NY 10065, USA; 4 College of Public Health, Hamad Bin Khalifa University, Qatar Foundation, Education City, Doha 24144, Qatar

**Keywords:** HIV, serodiscordancy, geographical clustering, sub Saharan Africa

## Abstract

Introduction: Variation in the proportion of individuals living in a stable HIV sero-discordant partnership (SDP), and the potential drivers of such variability across sub Saharan Africa (SSA), are still not well-understood. This study aimed to examine the spatial clustering of HIV sero-discordancy, and the impact of local variation in HIV prevalence on patterns of sero-discordancy in high HIV prevalence countries in SSA. Methods: We described the spatial patterns of sero-discordancy among stable couples by analyzing Demographic and Health Survey data from Cameroon, Kenya, Lesotho, Tanzania, Malawi, Zambia, and Zimbabwe. We identified spatial clusters of SDPs in each country through a Kulldorff spatial scan statistics analysis. After a geographical cluster was identified, epidemiologic measures of sero-discordancy were calculated and analyzed. Results: Spatial clusters with significantly high numbers of SDPs were identified and characterized in Kenya, Malawi, and Tanzania, and they largely overlapped with the clusters with high HIV prevalence. There was a positive correlation between HIV prevalence and the proportion of SDPs among all stable couples across within and outside clusters. Conversely, there was a negative, but weak and not significant, correlation between HIV prevalence and the proportion of SDPs among all stable couples with at least one HIV-infected individual in the partnership. Discussion: There does not appear to be distinct spatial patterns for HIV sero-discordancy that are independent of HIV prevalence patterns. The variation of the sero-discordancy measures with HIV prevalence across clusters and outside clusters demonstrated similar patterns to those observed at the national level. The spatial variable does not appear to be a fundamental nor independent determinant of the observed patterns of sero-discordancy in high HIV prevalence countries in SSA.

## 1. Introduction

The variation in the proportion of individuals living in a stable HIV sero-discordant partnership (SDPs; i.e., one partner testing HIV seropositive, while the other testing HIV seronegative) [1,2,3], and the potential drivers of variability in HIV sero-discordancy patterns across sub Saharan Africa (SSA) are still not well understood. There is also an intense discussion regarding the potential role of HIV seroconversions among SDPs in the HIV epidemic in SSA, and the prioritization of HIV control interventions focused on HIV discordant couples over other intervention approaches [4,5,6,7,8].

A number of studies have assessed the dynamics of HIV discordancy among SDPs and contribution of SDPs to HIV epidemics, using the cross-sectional Demographic and Health Survey (DHS) data as input data [9], and found that HIV epidemiology among heterosexual stable couples appears to be a predictable “spill-over” effect of the “core” HIV dynamics in the population [1,8,10,11,12,13]. Moreover, the studies indicated that national-level HIV prevalence explains most of the variation in HIV discordancy measures [1,11]. These studies also found that in HIV high-prevalence countries, a considerable proportion of stable couples were affected by HIV, and half of these stable couples were HIV discordant [1,11]. In contrast, in low-prevalence countries, smaller proportions of stable couples were affected by HIV, but a higher fraction of these couples were HIV discordant [1,11]. These studies concluded that for most countries, HIV incidence among SDPs is unlikely to exceed 50% of new HIV infections in the population at large [8,13].

While these studies included data from 20 countries from SSA, estimations were conducted using aggregated data at the national level [1,8,10,11,12,13]. Recent studies have shown stark geographical variation in HIV prevalence at the subnational level in most countries in SSA, in which a geographical clustered HIV transmission within micro-epidemics of different scales appears to be a common pattern [14]. This local variation in HIV prevalence within a country may reflect the spatial and temporal dynamics of the HIV epidemic in the country [15], and estimations using aggregated data at national level could mask local dynamics of the infection. Consequently, an assessment of the geographical patterns of HIV discordancy is of particular relevance.

Against this background, and with the ultimate goal of developing more effective strategies for HIV prevention interventions, this study aimed to (1) examine the geographical distribution of HIV sero-discordancy at the country level and locate clusters of high numbers of SDPs; (2) characterize these clusters by estimating key measures of sero-discordancy; (3) explore descriptively the overlap between SDP clusters and HIV infection clusters; and (4) assess the association between several sero-discordancy measures and HIV prevalence across identified clusters and outside identified clusters. Accordingly, our study aims to clarify HIV epidemiology at the intersection of two topical themes of HIV research today, spatial dimension of the distribution of HIV infection and the role of SDPs in the HIV epidemics in SSA.

## 2. Methods

### 2.1. Data

We described the geographical patterns of HIV infection among stable couples in countries with high HIV prevalence in SSA by analyzing the DHS data [9]. A stable couple was defined, per DHS methodology, as a man and a woman living together in a consensual union within a household at the time of the cross-sectional DHS survey [9]. Every couple in a polygamous partnership was considered as a separate union. An age-based definition for the sexually active population was used including women aged 15–49 years, and men aged 15–49, or 15–54, or 15–59 years, depending on the inclusion criteria used in each of the DHS surveys conducted in each country [9]. The DHS couple data set for each country included in the study was merged with the corresponding HIV serological biomarker data set. Couples where only one of the partners had been tested for HIV were excluded from the final analyses.

Since we aimed to investigate the geographical patterns of discordancy in countries with high HIV prevalence, countries with available DHS HIV serological biomarker survey and with HIV prevalence larger than 5% were included in the analyses. As a result, a total of seven countries in SSA were included: Cameroon (2004), Kenya (2008–2009), Lesotho (2009), Tanzania (2007–2008), Malawi (2010), Zambia (2007), and Zimbabwe (2010–2011).

### 2.2. Spatial Clustering Detection

We identified spatial clusters with high numbers of HIV sero-discordant couples in each country through a Kulldorff spatial scan statistics analysis [16]. This methodology is one the most widely used tests for clustering detection in epidemiology [17,18,19,20,21]. Spatial scan statistics is based on a cluster detection test which has been designed to identify the location of areas with higher numbers of cases (i.e., HIV discordant couples) than it would be expected under the assumption of random distribution of cases in space, and then evaluate their statistical significance by gradually scanning a circular window that scans across the entire study region. The radius of the circle is continuously changing, and it can take any value from 0 up to a pre-specified maximum value. For our analyses, a maximum circular window of a 100 km radius was used for scanning potential clusters with high numbers of HIV SDPs, as informed by a previous analysis [14].

The statistical significance of each potential cluster was estimated using a likelihood ratio test. This test is computed assuming a Bernoulli model with 0/1 event data for cases and non-cases [16]. A null hypothesis of spatial randomness is used to compare the numbers of observed and expected HIV sero-discordant couples within and outside the circular window. Circular windows with the highest likelihood ratio values were identified as potential clusters. Geographical clusters with a *p* < 0.05, calculated through Monte Carlo simulations (using the default value of 999 Monte Carlo simulations), were identified as statistically significant, and were further analyzed for additional epidemiological description. Kulldorff spatial scan statistics analysis was conducted using the software SatScan version 9.4.2 (Harvard Medical School, Boston, MA, USA), a software that examines clustering using state-of-the-art methodological and computational approaches, and accounts for recent theoretical progress in the field of spatial scan statistics [16].

### 2.3. Cluster Characterization and Correlations

After a geographical cluster with high number of SDPs was identified, sero-discordancy was characterized using several established population-level epidemiological measures [1]. Table 1 summarizes the estimated measures along with their epidemiological description. These measures were also estimated in high HIV prevalence clusters that were identified in our previous work [14]. Briefly, $P_{all}$ quantifies the proportion of discordant partnerships out of all stable sexual partnerships in the population, thereby conveying the scale of discordancy among stable partnerships in a population. It is also the measure most closely related to SDP cluster identification, and therefore SDP clusters could be seen effectively as clusters of high $P_{all}$. $P_{discord}$ estimates the proportion of discordant partnerships out of all partnerships in which at least one partner is HIV infected. Accordingly, it conveys the proportion of HIV-affected partnerships in which the uninfected partner has not yet acquired the infection from the infected partner, or from an external source. The complement of this measure, $1-P_{discord}$, provides the proportion of HIV-affected partnerships in which both partners are infected. $P_{all}$ quantifies the proportion of individuals that are part of a discordant partnership out of all sexually active individuals. Accordingly, it conveys the abundance of individuals that are engaged in discordant partnerships in a population. $I_{pos}$ estimates the proportion of all HIV infected persons that are engaged in a discordant partnership. Accordingly, it conveys the level of engagement of HIV positive persons in discordant partnerships at a specific time.

Each of these discordancy measures highlights a different epidemiological aspect of discordancy in a given population. $P_{all}$ and $I_{all}$ map the presence of discordancy among partnerships and sexually active individuals, respectively, while $I_{pos}$ maps the presence of discordancy among HIV infected persons. $P_{discord}$ underlines the efficiency of HIV transmission, and the potential for further HIV transmission, among discordant partners. All of these measures are cross-sectional in nature defined on a given cross-sectional survey at a specific time. Therefore, none of them captures how the partnership became a discordant partnership, whether it was formed between an HIV positive person and an HIV negative person, or one of the partners acquired the infection through extra-marital sex after formation of the partnership.

Correlations between these measures and HIV prevalence across the identified clusters and outside clusters were determined using Pearson correlation coefficient (PCC). Statistical analyses were conducted using SAS version 9.3 (SAS Institute Inc., Cary, NC, USA) [22], and all geographic information system (GIS) analyses and cartographic displays were performed with the software ArcGIS version 9.2 (ESRI, Redlands, CA, USA) [23].

## 3. Results

### 3.1. Spatial Clustering of SDPs and HIV Prevalence

**Figure 1:**
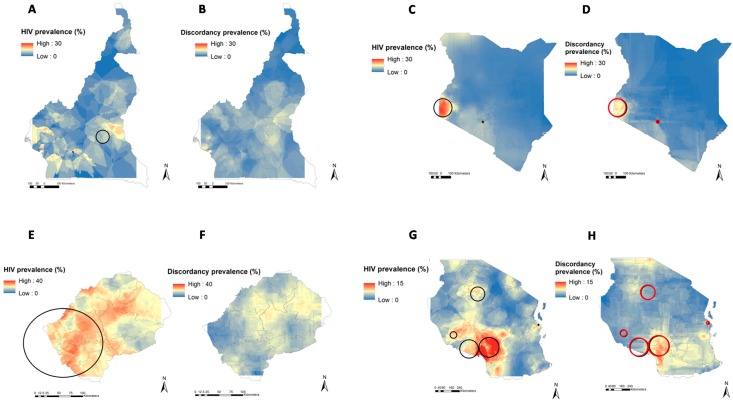

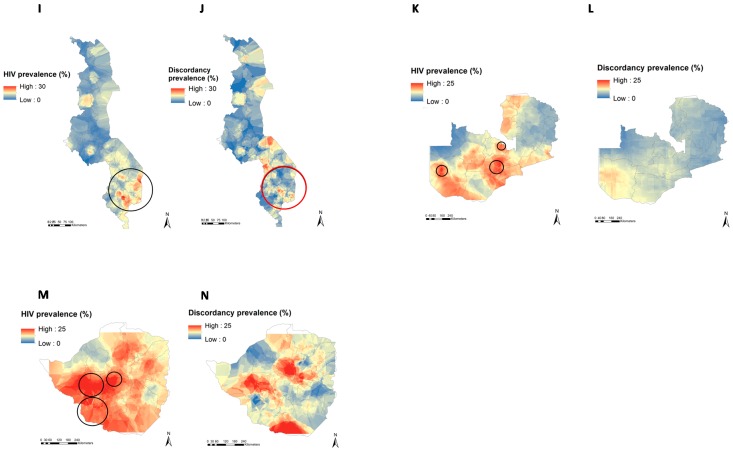
Geographical clustering of the number of HIV infections and the number of HIV sero-discordant partnerships in Cameroon, Kenya, Lesotho, Tanzania, Malawi, Zambia, and Zimbabwe. Black circles delineate spatial locations of high HIV prevalence clusters and red circles delineates high HIV SDP clusters in Cameroon (**A**,**B**), Kenya (**C**,**D**), Lesotho (**E**,**F**), Tanzania (**G**,**H**), Malawi (**I**,**J**), Zambia (**K**,**L**), and Zimbabwe (**M**,**N**). Continuous surfaces of HIV prevalence (**A**,**C**,**E**,**G**,**I**,**K**,**M**) and sero-discordant partnership prevalence (**B**,**D**,**F**,**H**,**J**,**L**,**N**) within a country were generated using the inverse distance weighted mapping algorithm [23].

From the 17,863 couples sampled in the seven countries, 16,140 (90.04%) had HIV biomarker collection for both individuals, and were included in our analyses. Figure 1 illustrates the location of the clusters with high numbers of HIV SDPs, and clusters with high HIV prevalence in each of the countries included in our study. Spatial clusters with significantly high numbers of SDPs were identified in Kenya, Malawi and Tanzania, and they largely overlapped with the clusters with high HIV prevalence. Even in countries or regions where no statistically significant SDP clusters could be identified, there was tendency for SDP clustering that strongly overlapped with HIV clustering (Figure 1). Therefore, the remaining analyses and cluster characterization were conducted using the clusters with high HIV prevalence. The locations and epidemiological characteristics of these spatial clusters with high HIV prevalence have been described elsewhere [14]. Briefly, SatScan identified five clusters with high HIV prevalence in Tanzania, two in Cameroon, one in Malawi, two in Kenya, three in Zambia, one in Zimbabwe, and three in Lesotho. HIV prevalence within these clusters ranged from 9% in Tanzania to 26% in Lesotho.

### 3.2. Measures of HIV Sero-Discordancy within and Outside of Clusters with High HIV Prevalence

**Figure 2:**
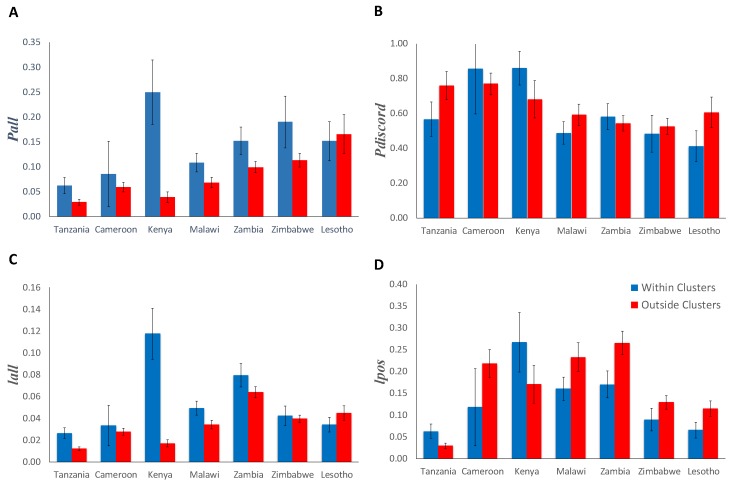
Epidemiological measures of sero-discordancy within and outside of clusters with high HIV prevalence in each of the countries included in the study. (**A**) The proportion of stable discordant partnerships among all stable partnerships ($P_{all}$); (**B**) the proportion of HIV discordant partnerships among all stable partnerships with at least one HIV-infected individual in the partnership ($P_{discord}$); (**C**) the proportion of individuals engaged in stable HIV discordant partnerships ($I_{all}$); (**D**) the proportion of all HIV-infected individuals engaged in stable HIV discordant partnerships ($I_{pos}$). Countries are shown in order of increasing national HIV prevalence.

Table 2 and Figure 2 summarizes the epidemiological measures for HIV sero-discordancy within and outside of clusters with high HIV prevalence in each of the countries included in the analyses. $P_{all}$ was highest within clusters with high HIV prevalence in Kenya (0.25) and lowest outside clusters with high HIV prevalence in Tanzania (0.03). Likewise, $P_{discord}$ was highest within clusters of high HIV prevalence in Kenya (0.86) and lowest outside clusters of high HIV prevalence in Zimbabwe (0.52). $I_{all}$ and $I_{pos}$ were also highest within clusters with high HIV prevalence in Kenya (0.12 and 0.27, respectively), whereas $I_{all}$ and $I_{pos}$ were lowest outside of clusters with high HIV prevalence in Tanzania (0.011) and Lesotho (0.11), respectively.

Epidemiological measures for HIV sero-discordancy ($P_{all}$, $P_{discord}$, $I_{all}$, $I_{pos}$) differed among countries, and within and outside of clusters with high HIV prevalence (Figure 2). Estimations of $P_{all}$ were significantly higher within clusters with high HIV prevalence in most of the countries with the exception of Cameroon (*p* = 0.41) and Lesotho (*p* = 0.64) (Figure 2A). In Kenya, measures for $P_{all}$ were considerably higher within clusters (0.25) compared to outside of the clusters with high HIV prevalence (0.04). Conversely, measures for $P_{discord}$ within and outside of the clusters with high HIV prevalence were not significantly different in any of the countries included in the analysis (Figure 2B). Estimations of $I_{all}$ were significantly higher within clusters of high HIV prevalence in Tanzania, Kenya, Malawi, and Zambia (*p* < 0.05), but they were not significantly different within compared to outside clusters with high HIV prevalence in Cameroon (*p* = 0.54), Zimbabwe (*p* = 0.51), and Lesotho (*p* = 0.53) (Figure 2C). Lastly, measures for $I_{pos}$ were significantly lower within clusters with high HIV prevalence in Malawi, Zambia, Zimbabwe, and Lesotho (*p* < 0.05), but they were not significantly different in Tanzania (*p* = 0.09), Cameroon (*p* = 0.15), and Kenya (*p* = 0.07) (Figure 2D).

### 3.3. Associations between Measures of Sero-Discordancy and HIV Prevalence

**Figure 3:**
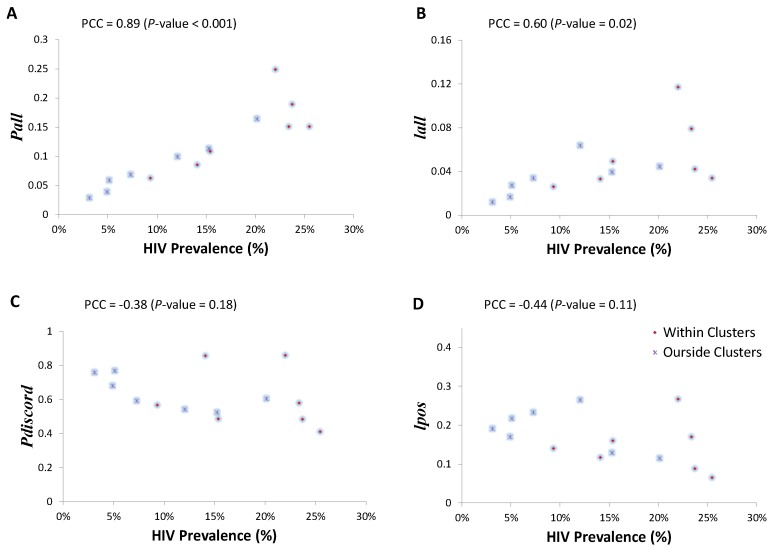
Associations between measures of sero-discordancy and HIV prevalence. (**A**) Scatter plot of the proportion of stable discordant couples among all stable couples ($P_{all}$) versus HIV prevalence within and outside the high HIV prevalence clusters; (**B**) scatter plot of the proportion of individuals engaged in stable HIV discordant couple ($I_{all}$) versus HIV prevalence within and outside the high HIV prevalence clusters; (**C**) scatter plot of the proportion of HIV discordant couples among all stable couples with at least one HIV-infected individual in the couple ($P_{discord}$) versus HIV prevalence within and outside the high HIV prevalence clusters; (**D**) scatter plot of the proportion of all HIV-infected individuals engaged in stable HIV discordant partnerships ($I_{pos}$) versus HIV prevalence within and outside the high HIV prevalence clusters. Correlations were determined using Pearson correlation coefficient (PCC).

There was a positive correlation between HIV prevalence and $P_{all}$ (PCC = 0.89; 95% Confidence Interval (CI) 0.68–0.96), and HIV prevalence and $I_{all}$ (PCC = 0.60; 95% CI 0.1–0.86) across within and outside HIV prevalence clusters (Figure 3A,B). Conversely, there was a negative, but weak and not significant, correlation between HIV prevalence and $P_{discord}$ (PCC = −0.38; 95% CI −0.75–0.18), and HIV prevalence and $I_{pos}$ (PCC = −0.44; 95% CI −0.79–0.11) across within and outside HIV prevalence clusters (Figure 3C,D).

## 4. Discussion

We characterized the spatial distribution and clustering of SDPs in high HIV prevalence countries in SSA. We also presented different epidemiologic measures of sero-discordancy within and outside HIV infection clusters and assessed the association between these measures and HIV prevalence. Our results suggest that there are no distinct spatial patterns for HIV sero-discordancy that are independent of HIV prevalence patterns. The spatial clusters of discordancy overlapped with those for HIV prevalence and there were no discordancy clusters that are distinct from those of HIV prevalence.

The variation of the epidemiological measures of discordancy with HIV prevalence across clusters and outside clusters demonstrated similar patterns to those observed at the national level [1]. The discordancy patterns seen at the national level were mirrored at the sub-national cluster level with no signature for an independent role for geographic variation. This finding is consistent with the picture emerging form a series of studies on discordancy [1,8,10,11,12,13] that HIV epidemiology among heterosexual stable couples appears to be simply a predictable “spill-over” effect of the “core” HIV dynamics in the population, and that stable discordant couples do not constitute a core factor in dictating the dynamics of HIV infection.

$P_{all}$ and $I_{all}$ were found to significantly increase with higher HIV prevalence at local level, just as they do at the national level [1]. As the local HIV prevalence increases, the number of HIV infected individuals increases as well, and therefore more discordant partnerships are likely to form in the population, and larger fraction of the sexually active population would be part of a discordant partnership. The observed correlations between both $P_{all}$ and $I_{all}$ and HIV prevalence affirm this explanation and that the dynamics of discordancy, whether within or outside clusters, just mirrored the dynamics in the whole population.

Though the trend was not significant for $P_{discord}$ and $I_{pos}$, both of these measures of discordancy were found to decline with higher HIV prevalence, also just as observed at the national level [1]. These measures describe the persistence of discordancy among HIV-affected partnerships, that is, the chance that these partnerships will form and will remain sero-discordant as opposed to sero-concordant positive. With higher HIV prevalence, there are more chances for an HIV-affected partnership to be or to become concordant positive as opposed to discordant. Therefore, this pattern further affirms similarity of HIV discordancy dynamics whether within or outside clusters, or in the whole population.

The above results are also consistent with the national analyses indicating that most of the variability in discordancy measures could be explained by HIV prevalence alone [1,11]. Kenya provided a stark example to this end where the discordancy measures varied immensely between within and outside clusters, mirroring the stark variation in HIV infection distribution in this country. Our results also affirm that discordant couples constitute the majority of stable couples where at least one partner is HIV infected ($P_{discord}$) in geographic areas with low HIV prevalence; conversely, only half of these stable couples are discordant in areas with high HIV prevalence. Moreover, the fraction of couples engaged in an SDP is low in areas with low HIV prevalence, and high in areas with high HIV prevalence.

Our findings suggest that the spatial dimension does not appear to be a fundamental nor independent determinant of the observed patterns of sero-discordancy in high HIV prevalence countries in SSA. While the spatial dimension may not be critical for a proper understanding of discordancy dynamics, the temporal dimension could be an important factor, and may influence discordancy as an HIV epidemic sweeps through a population. Interventions, such as antiretroviral therapy, may also influence the dynamics of discordancy and contribution of SDPs to HIV incidence. Investigating the time variable in discordancy dynamics may provide important insights about drivers of the historical evolution of HIV epidemics, and future projections for discordancy and for SDPs’ role in the epidemic.

Our study has several limitations. We did not find statistically significant SDP clusters in several countries. However, this is likely a consequence of the fact that the number of SDPs is smaller than the number of HIV infections in any region, leading to lack of statistical power to detect these clusters. Moreover, even when there were no statistically significant SDP clusters, there was a trend for SDP clustering. This limitation therefore is not likely to have affected our conclusions. Inclusion of countries in this study was constrained by the availability of a DHS with HIV biomarker information and geographical coordinates of each survey data point. While DHS data are of high quality, they may not be statistically powered to answer research questions at the identified sub-national clusters level. This has limited our ability to control for potential biological or behavioral confounders that could be associated with SDP or HIV clustering besides HIV prevalence. Further analyses including surveys statistically powered in small geographic areas may be necessary to identify the drivers of SDP and HIV spatial clustering in SSA.

Despite the fact that the DHS program has a detailed and well-validated protocol to conduct HIV biomarker data collection, laboratory testing of surveyed specimens is conducted in the host country and may vary from one country to another. Specific assays used in the HIV testing algorithm are generally determined in coordination with the host country, which could be a source of data variation and quality between countries. It has been highlighted recently, based on an analysis of 20 countries, that testing errors associated with false positivity could be present in DHS surveys [24]. Substantial variation among countries was observed, with greater magnitude of error introduced by false HIV positive results in Malawi, Niger, Sierra Leone, Senegal, and Zambia. Although we conducted our analyses in several countries at the same time, and despite the fact that there are not clear conclusions regarding the impact of this source of error on HIV prevalence estimations and other type of analyses including HIV biomarkers, it is prudent to take into consideration this source of error and interpret our results within the context of this limitation.

Lastly, given the multiple logistical difficulties in conducting DHS rounds, some of our measures could have been influenced by inherent biases in the data such as the variability in response rate to HIV testing [25,26]. A potential bias in our study is the geographical position system (GPS) displacement process of the DHS sampling data points, used to preserve the confidentiality of the data points [27], which could have impacted the precision of the geographical location of the discordancy and HIV prevalence clusters (by few kilometers at most).

## 5. Conclusions

Discordant couples are a key population for HIV prevention programs [1,2,3] and several prevention interventions have been developed and introduced to benefit this population group [4,5,6,7,8]. While it is important to target this population with interventions, it should be factored that HIV dynamics among SDPs is probably just a spill-over effect of the core HIV dynamics in the population. Controlling the HIV epidemic requires addressing the core drivers of infection transmission in the population, such as among commercial sex networks.

## Figures and Tables

**Table 1:** Population-level epidemiological measures of HIV sero-discordancy [1].

Measure	Definition	Estimation	Description
$P_{all}$	Proportion of SDPs * among all stable couples	$\frac{\text{number of stable HIV discordant partnerships}}{\text{total number of stable partnerships}}$	Measures the level of sero-discordancy among all stable couples in the population
$P_{discord}$	Proportion of SDPs among all stable couples with at least one HIV-infected individual in the partnership	$\frac{\text{number of stable HIV discordant partnerships}}{\text{total number of stable partnerships with at least one HIV infected individual}}$	Measures the proportion of stable couples affected by HIV where the uninfected partner has not acquired the infection but is at risk of acquiring it from the infected partner
$I_{all}$	Proportion of individuals engaged in SDPs among the entire sexually active population	$\frac{\text{number of individuals in stable HIV discordant partnerships}}{\text{total number of individuals}}$	Measures the abundance of individuals who are engaged in SDPs in the population
$I_{pos}$	Proportion of HIV-infected individuals engaged in SDPs among the entire HIV-infected population	$\frac{\text{number of HIV infected individuals in stable HIV discordant partnerships}}{\text{total number of HIV infected individuals}}$	Measures the level of engagement of HIV infected individuals in SDPs

* SDP: Sero-discordant partnership.

**Table 2:** Epidemiological measures of HIV sero-discordancy within and outside of clusters with high HIV prevalence in each of the countries included in the study.

Country	Within Clusters with High HIV Prevalence					Outside Clusters with High HIV Prevalence				
	HIV Prevalence (%)	$P_{all}$ *	$P_{discord}$ ^+^	$I_{all}$ ^#^	$I_{pos}$ ^^^	HIV Prevalence (%)	$P_{all}$ *	$P_{discord}$ ^+^	$I_{all}$ ^#^	$I_{pos}$ ^^^
**Tanzania**	9.33	0.063	0.567	0.026	0.140	3.11	0.029	0.759	0.011	0.191
**Cameroon**	14.10	0.086	0.857	0.033	0.117	5.10	0.059	0.769	0.027	0.217
**Malawi**	15.38	0.108	0.487	0.049	0.159	7.29	0.068	0.591	0.034	0.232
**Kenya**	22.02	0.250	0.860	0.117	0.267	4.90	0.039	0.680	0.016	0.170
**Zambia**	23.38	0.152	0.580	0.079	0.169	12.06	0.099	0.542	0.063	0.264
**Zimbabwe**	23.73	0.190	0.482	0.042	0.088	15.26	0.113	0.524	0.039	0.129
**Lesotho**	25.48	0.152	0.412	0.034	0.065	20.14	0.165	0.605	0.044	0.114

* Proportion of stable discordant partnerships among all stable partnerships; ^+^ Proportion of HIV discordant partnerships among all stable partnerships with at least one HIV-infected individual in the partnership; ^#^ Proportion of individuals engaged in stable HIV discordant partnerships; ^^^ Proportion of all HIV-infected individuals engaged in stable HIV discordant partnerships.
